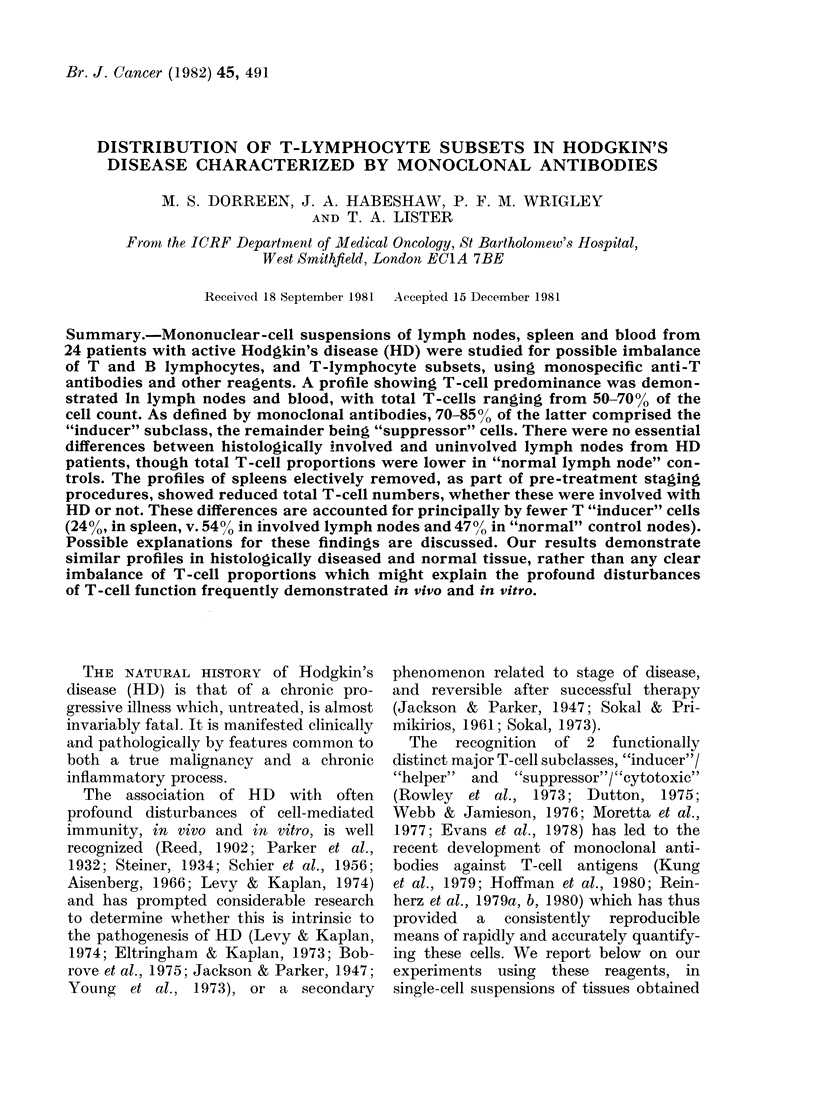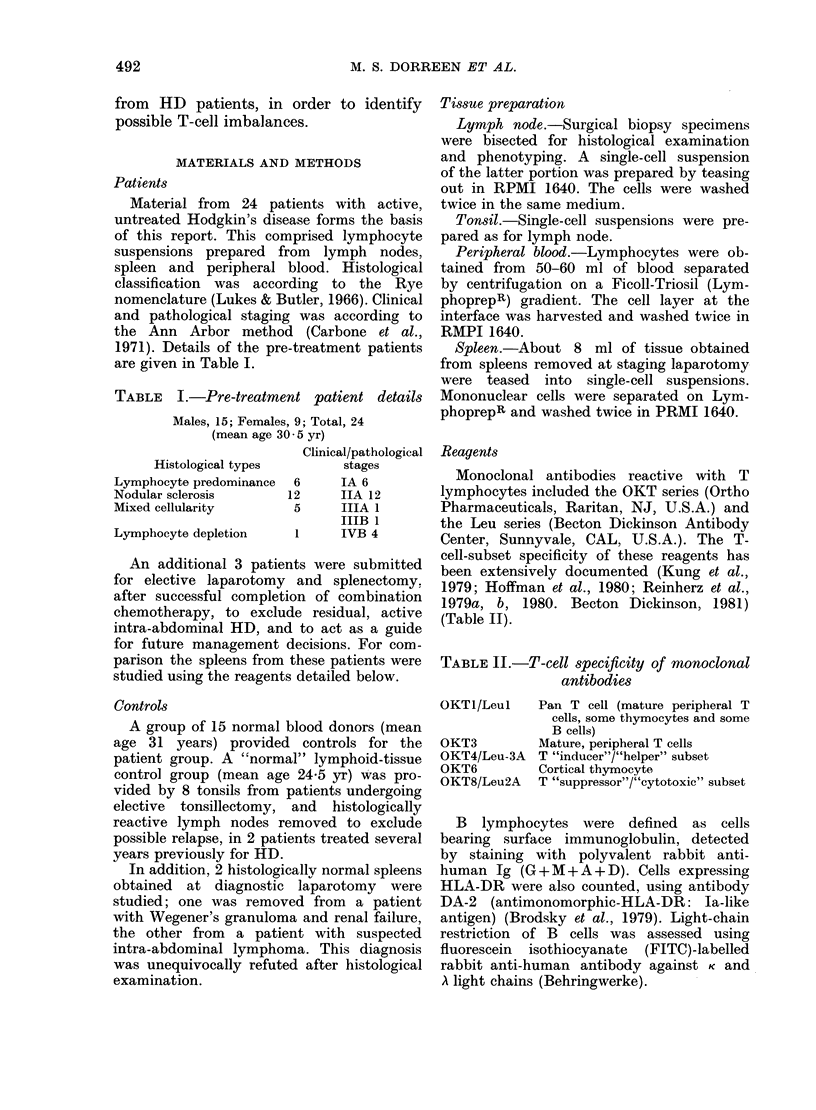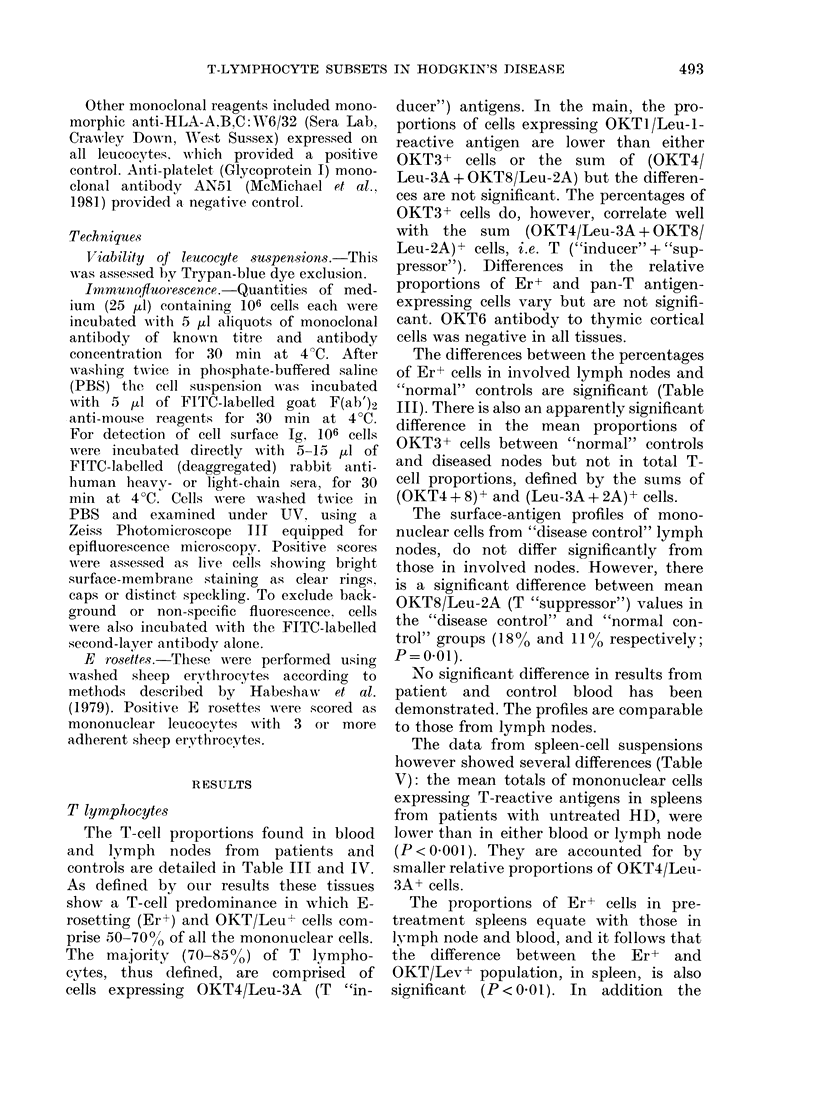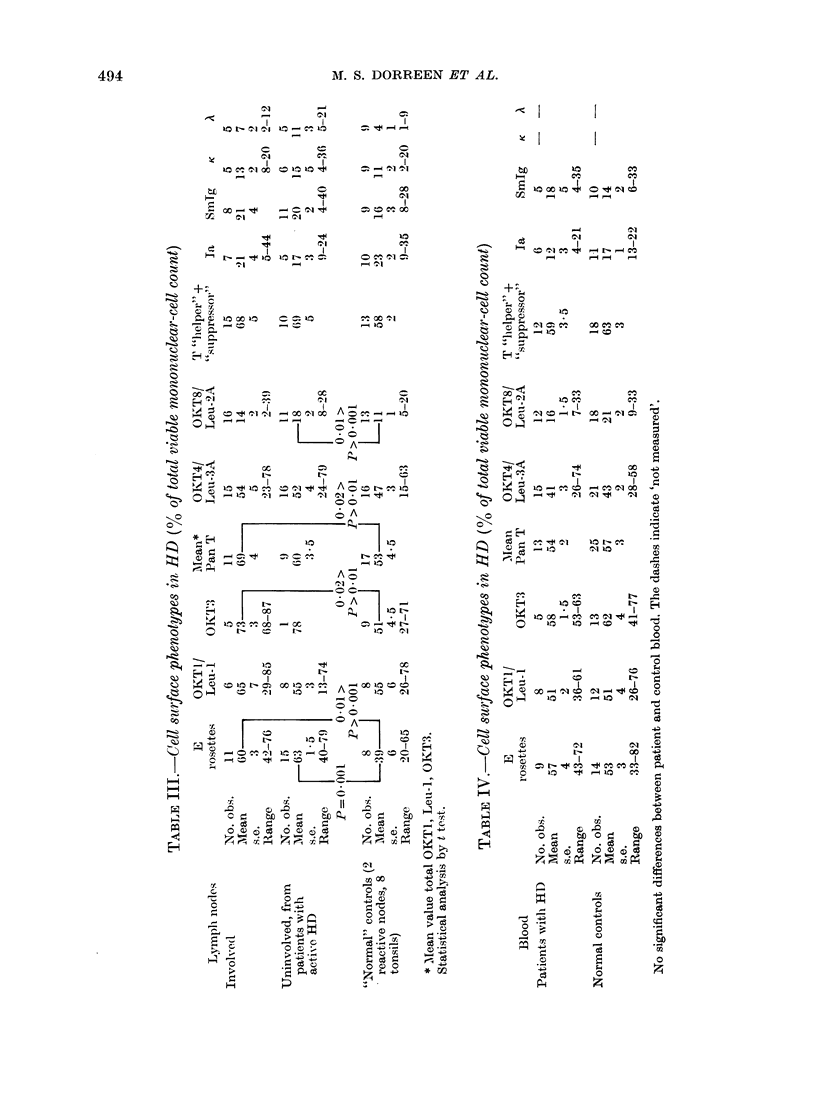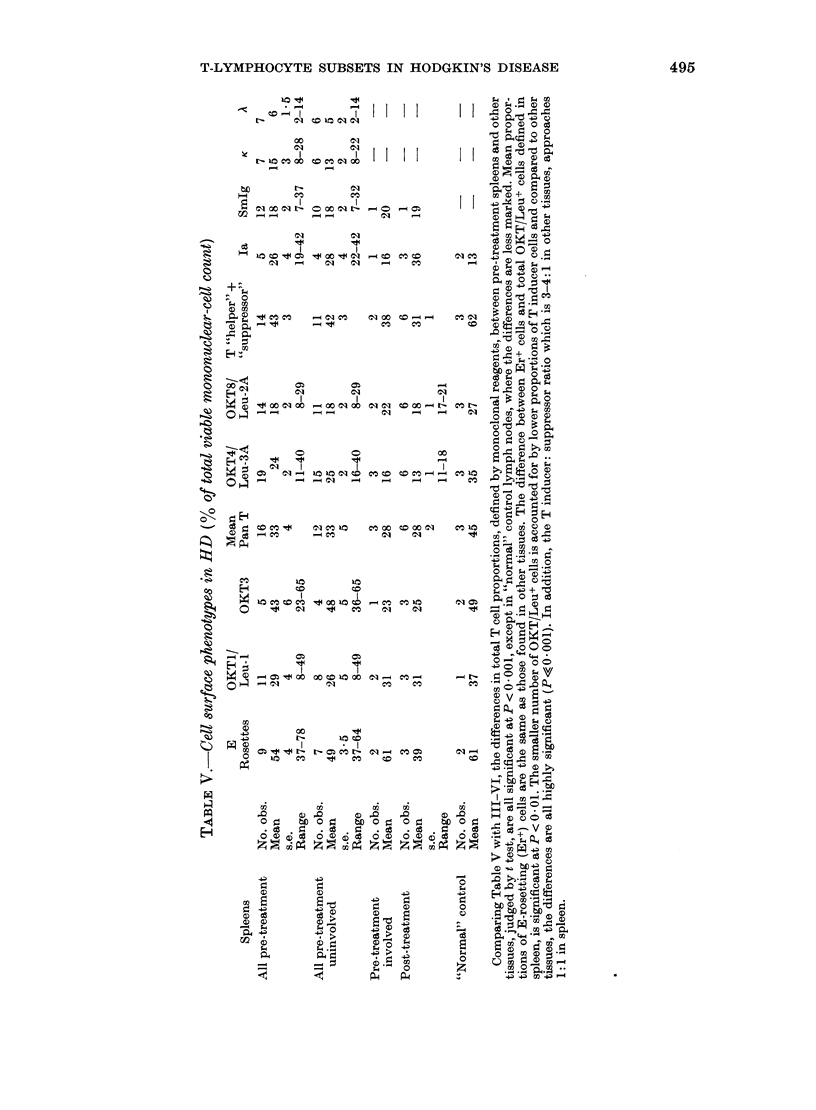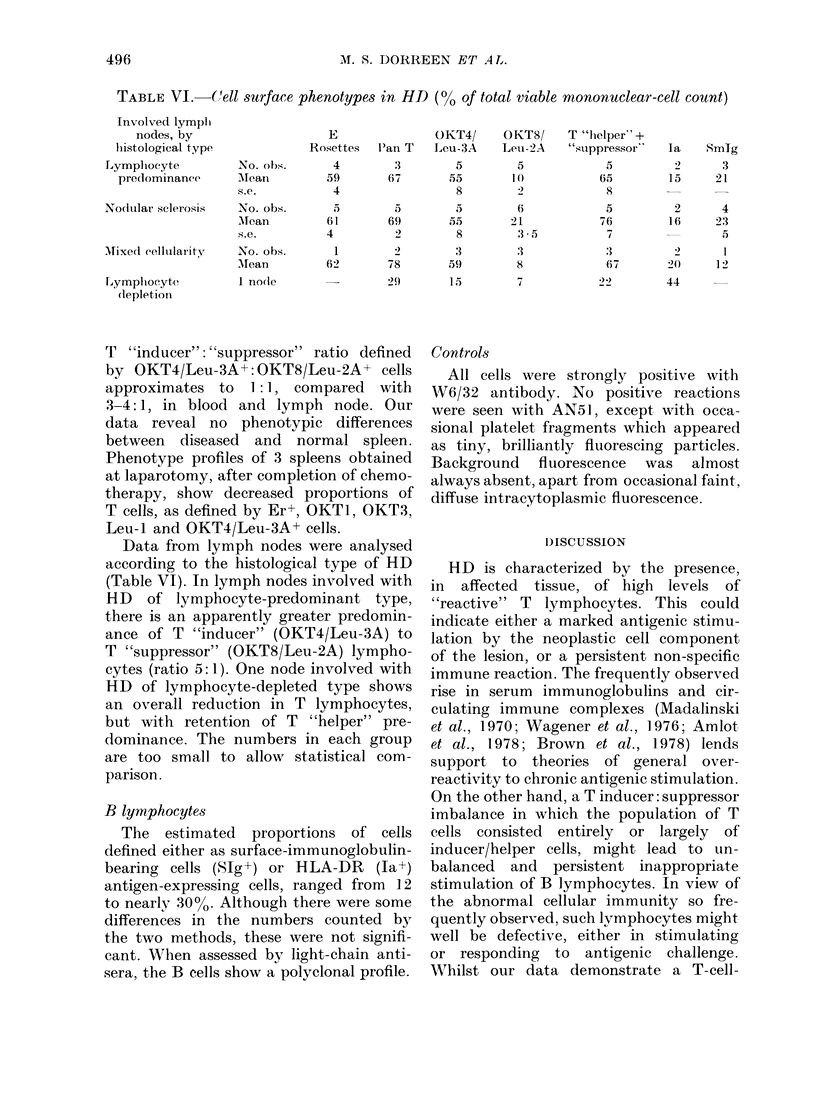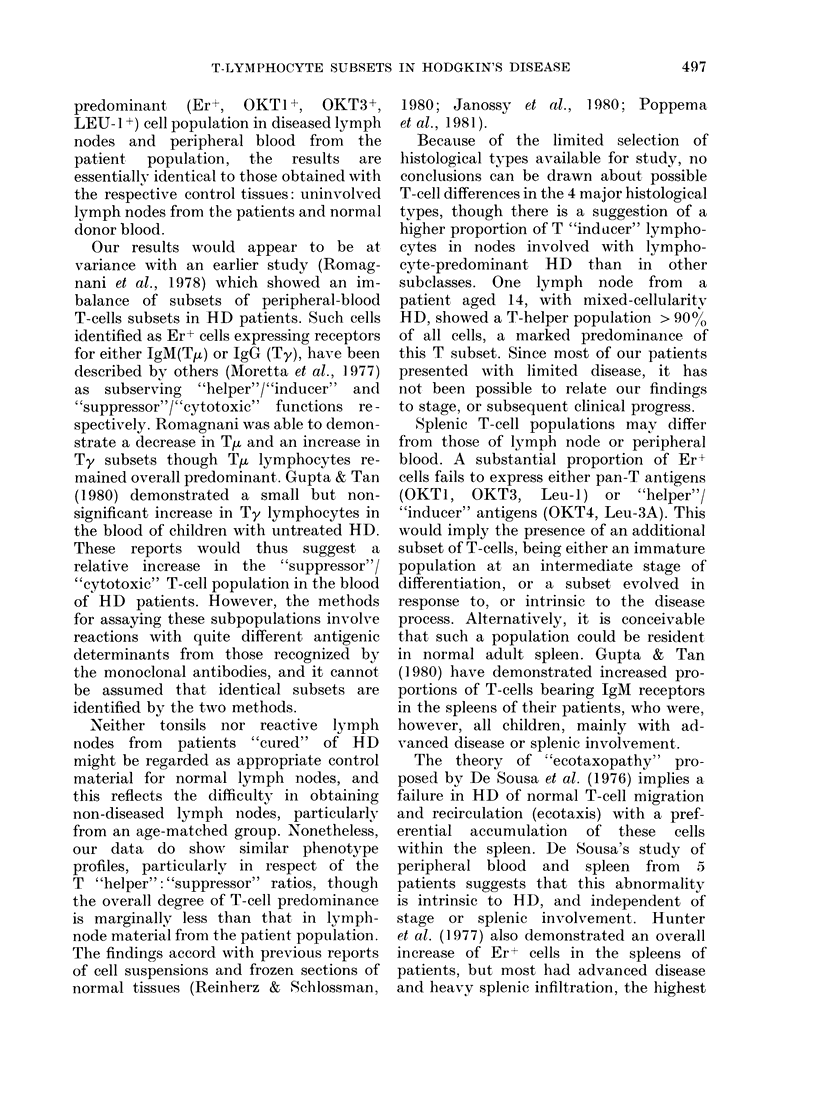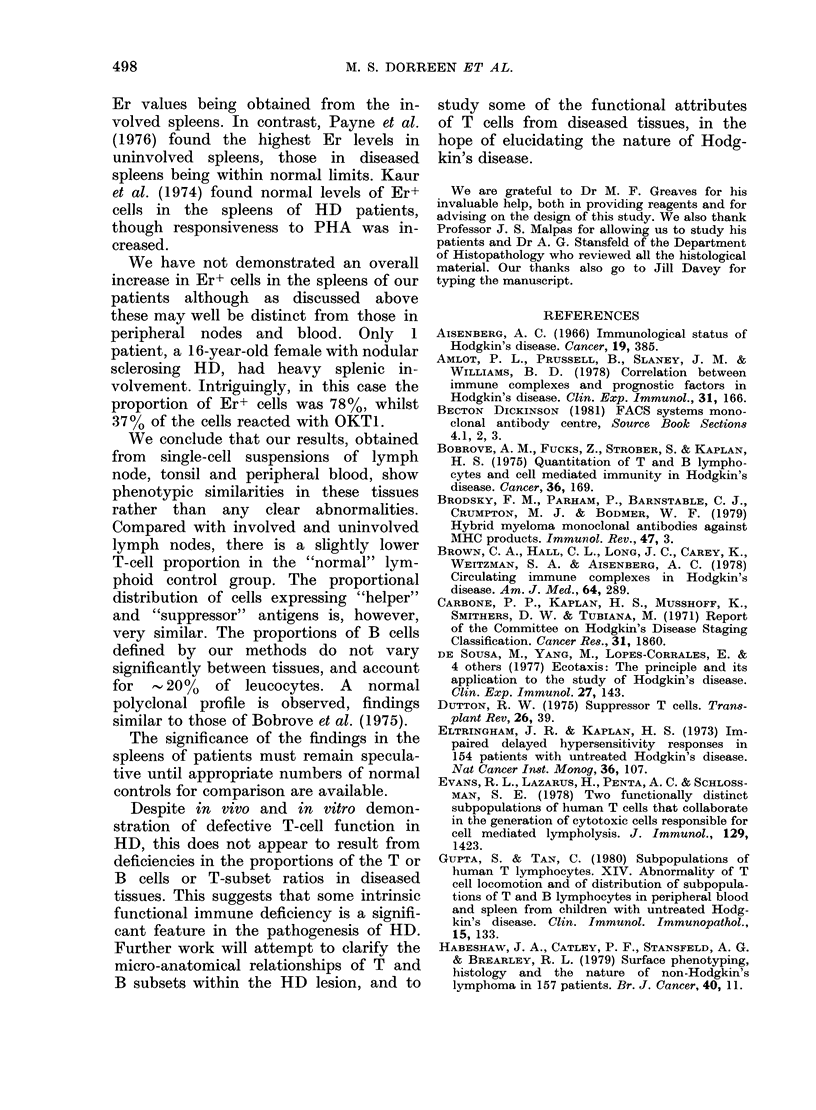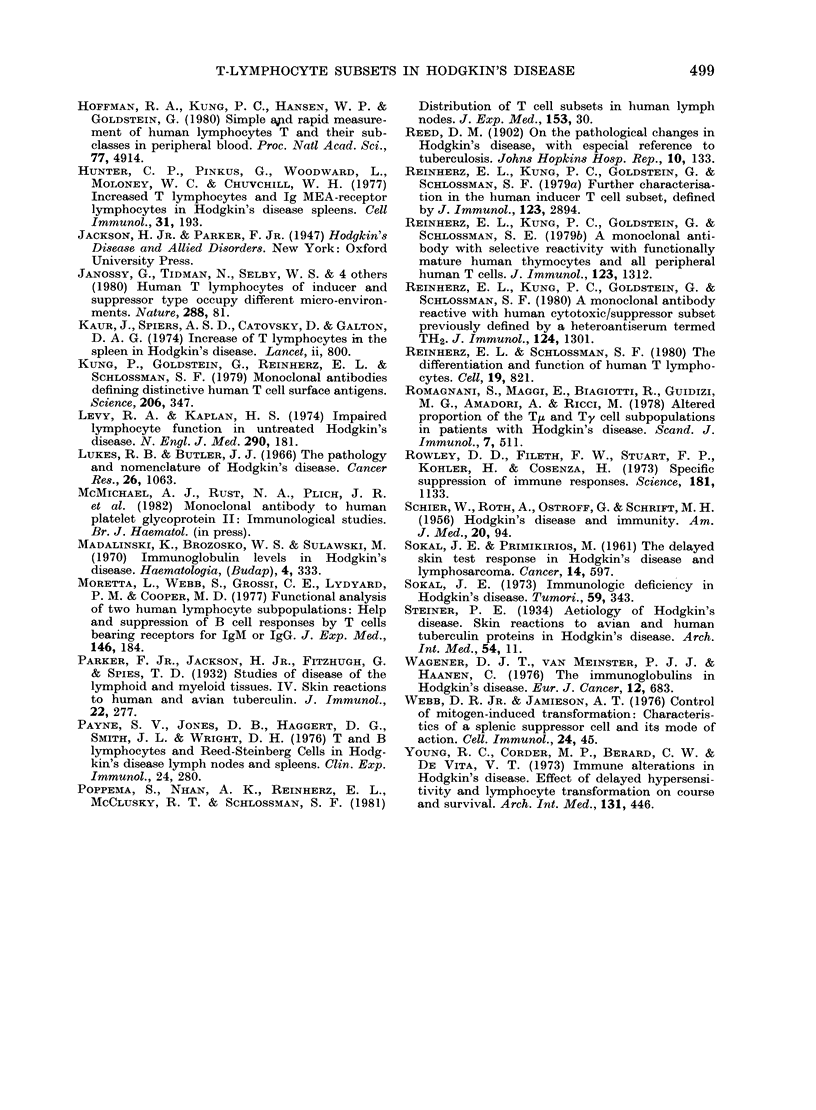# Distribution of T-lymphocyte subsets in Hodgkin's disease characterized by monoclonal antibodies.

**DOI:** 10.1038/bjc.1982.84

**Published:** 1982-04

**Authors:** M. S. Dorreen, J. A. Habeshaw, P. F. Wrigley, T. A. Lister

## Abstract

Mononuclear-cell suspensions of lymph nodes, spleen and blood from 24 patients with active Hodgkin's disease (HD) were studied for possible imbalance of T and B lymphocytes, and T-lymphocyte subsets, using monospecific anti-T antibodies and other reagents. A profile showing T-cell predominance was demonstrated in lymph nodes and blood, with total T-cells ranging from 50-70% of the cell count. As defined by monoclonal antibodies, 70-85 of the latter comprised the "inducer" subclass, the remainder being "suppressor" cells. There were no essential differences between histologically involved and uninvolved lymph nodes from HD patients, though total T-cell proportions were lower in "normal lymph node" controls. The profiles of spleens electively removed, as part of pre-treatment staging procedures, showed reduced total T-cell numbers, whether these were involved with HD or not. These differences are accounted for principally by fewer T "inducer" cells (24%, in spleen, v. 54% in involved lymph nodes and 47% in "normal" control nodes). Possible explanations for these findings are discussed. Our results demonstrate similar profiles in histologically diseased and normal tissue, rather than any clear imbalance of T-cell proportions which might explain the profound disturbances of T-cell function frequently demonstrated in vivo and in vitro.


*Br. J. Cancer* (1982) **45**, 491

### DISTRIBUTION OF T-LYMPHOCYTE SUBSETS IN HODGKIN'S DISEASE CHARACTERIZED BY MONOCLONAL ANTIBODIES

M. S. DORREEN, J. A. HABESHAW, P. F. M. WRIGLEY AND T. A. LISTER

*From the ICRF Department of Medical Oncology, St Bartholomew's Hospital, West Smithfield, London EC1A 7BE*

Received 18 September 1981 Accepted 15 December 1981

**Summary.—Mononuclear-cell suspensions of lymph nodes, spleen and blood from 24 patients with active Hodgkin's disease (HD) were studied for possible imbalance of T and B lymphocytes, and T-lymphocyte subsets, using monospecific anti-T antibodies and other reagents. A profile showing T-cell predominance was demonstrated ln lymph nodes and blood, with total T-cells ranging from 50–70% of the cell count. As defined by monoclonal antibodies, 70–85% of the latter comprised the "inducer" subclass, the remainder being "suppressor" cells. There were no essential differences between histologically involved and uninvolved lymph nodes from HD patients, though total T-cell proportions were lower in "normal lymph node" controls. The profiles of spleens electively removed, as part of pre-treatment staging procedures, showed reduced total T-cell numbers, whether these were involved with HD or not. These differences are accounted for principally by fewer T "inducer" cells (24%, in spleen, v. 54% in involved lymph nodes and 47% in "normal" control nodes). Possible explanations for these findings are discussed. Our results demonstrate similar profiles in histologically diseased and normal tissue, rather than any clear imbalance of T-cell proportions which might explain the profound disturbances of T-cell function frequently demonstrated *in vivo* and *in vitro*.**

THE NATURAL HISTORY of Hodgkin's disease (HD) is that of a chronic progressive illness which, untreated, is almost invariably fatal. It is manifested clinically and pathologically by features common to both a true malignancy and a chronic inflammatory process.

The association of HD with often profound disturbances of cell-mediated immunity, *in vivo* and *in vitro*, is well recognized (Reed, 1902; Parker *et al.*, 1932; Steiner, 1934; Schier *et al.*, 1956; Aisenberg, 1966; Levy & Kaplan, 1974) and has prompted considerable research to determine whether this is intrinsic to the pathogenesis of HD (Levy & Kaplan, 1974; Eltringham & Kaplan, 1973; Bobrove *et al.*, 1975; Jackson & Parker, 1947; Young *et al.*, 1973), or a secondary phenomenon related to stage of disease, and reversible after successful therapy (Jackson & Parker, 1947; Sokal & Primikirios, 1961; Sokal, 1973).

The recognition of 2 functionally distinct major T-cell subclasses, "inducer"/"helper" and "suppressor"/"cytotoxic" (Rowley *et al.*, 1973; Dutton, 1975; Webb & Jamieson, 1976; Moretta *et al.*, 1977; Evans *et al.*, 1978) has led to the recent development of monoclonal antibodies against T-cell antigens (Kung *et al.*, 1979; Hoffman *et al.*, 1980; Reinherz *et al.*, 1979*a*, *b*, 1980) which has thus provided a consistently reproducible means of rapidly and accurately quantifying these cells. We report below on our experiments using these reagents, in single-cell suspensions of tissues obtained from HD patients, in order to identify possible T-cell imbalances.

### MATERIALS AND METHODS

### *Patients*

Material from 24 patients with active, untreated Hodgkin's disease forms the basis of this report. This comprised lymphocyte suspensions prepared from lymph nodes, spleen and peripheral blood. Histological classification was according to the Rye nomenclature (Lukes & Butler, 1966). Clinical and pathological staging was according to the Ann Arbor method (Carbone *et al.*, 1971). Details of the pre-treatment patients are given in Table I.

TABLE I.—*Pre-treatment patient details*

Males, 15; Females, 9; Total, 24 (mean age 30·5 yr)

| Histological types | | Clinical/pathological stages |
|---|---|---|
| Lymphocyte predominance | 6 | IA 6 |
| Nodular sclerosis | 12 | IIA 12 |
| Mixed cellularity | 5 | IIIA 1 |
| | | IIIB 1 |
| Lymphocyte depletion | 1 | IVB 4 |

An additional 3 patients were submitted for elective laparotomy and splenectomy, after successful completion of combination chemotherapy, to exclude residual, active intra-abdominal HD, and to act as a guide for future management decisions. For comparison the spleens from these patients were studied using the reagents detailed below.

### *Controls*

A group of 15 normal blood donors (mean age 31 years) provided controls for the patient group. A "normal" lymphoid-tissue control group (mean age 24·5 yr) was provided by 8 tonsils from patients undergoing elective tonsillectomy, and histologically reactive lymph nodes removed to exclude possible relapse, in 2 patients treated several years previously for HD.

In addition, 2 histologically normal spleens obtained at diagnostic laparotomy were studied; one was removed from a patient with Wegener's granuloma and renal failure, the other from a patient with suspected intra-abdominal lymphoma. This diagnosis was unequivocally refuted after histological examination.

### *Tissue preparation*

*Lymph node.*—Surgical biopsy specimens were bisected for histological examination and phenotyping. A single-cell suspension of the latter portion was prepared by teasing out in RPMI 1640. The cells were washed twice in the same medium.

*Tonsil.*—Single-cell suspensions were prepared as for lymph node.

*Peripheral blood.*—Lymphocytes were obtained from 50–60 ml of blood separated by centrifugation on a Ficoll-Triosil (Lymphoprep$^R$) gradient. The cell layer at the interface was harvested and washed twice in RMPI 1640.

*Spleen.*—About 8 ml of tissue obtained from spleens removed at staging laparotomy were teased into single-cell suspensions. Mononuclear cells were separated on Lymphoprep$^R$ and washed twice in PRMI 1640.

### *Reagents*

Monoclonal antibodies reactive with T lymphocytes included the OKT series (Ortho Pharmaceuticals, Raritan, NJ, U.S.A.) and the Leu series (Becton Dickinson Antibody Center, Sunnyvale, CAL, U.S.A.). The T-cell-subset specificity of these reagents has been extensively documented (Kung *et al.*, 1979; Hoffman *et al.*, 1980; Reinherz *et al.*, 1979*a*, *b*, 1980. Becton Dickinson, 1981) (Table II).

TABLE II.—*T-cell specificity of monoclonal antibodies*

| | |
|---|---|
| OKT1/Leu1 | Pan T cell (mature peripheral T cells, some thymocytes and some B cells) |
| OKT3 | Mature, peripheral T cells |
| OKT4/Leu-3A | T "inducer"/"helper" subset |
| OKT6 | Cortical thymocyte |
| OKT8/Leu2A | T "suppressor"/"cytotoxic" subset |

B lymphocytes were defined as cells bearing surface immunoglobulin, detected by staining with polyvalent rabbit anti-human Ig (G+M+A+D). Cells expressing HLA-DR were also counted, using antibody DA-2 (antimonomorphic-HLA-DR: Ia-like antigen) (Brodsky *et al.*, 1979). Light-chain restriction of B cells was assessed using fluorescein isothiocyanate (FITC)-labelled rabbit anti-human antibody against $\kappa$ and $\lambda$ light chains (Behringwerke).

Other monoclonal reagents included monomorphic anti-HLA-A,B,C:W6/32 (Sera Lab, Crawley Down, West Sussex) expressed on all leucocytes, which provided a positive control. Anti-platelet (Glycoprotein I) monoclonal antibody AN51 (McMichael *et al.*, 1981) provided a negative control.

*Techniques*

*Viability of leucocyte suspensions.*—This was assessed by Trypan-blue dye exclusion.

*Immunofluorescence.*—Quantities of medium (25 $\mu$l) containing $10^6$ cells each were incubated with 5 $\mu$l aliquots of monoclonal antibody of known titre and antibody concentration for 30 min at 4°C. After washing twice in phosphate-buffered saline (PBS) the cell suspension was incubated with 5 $\mu$l of FITC-labelled goat $F(ab')_2$ anti-mouse reagents for 30 min at 4°C. For detection of cell surface Ig, $10^6$ cells were incubated directly with 5–15 $\mu$l of FITC-labelled (deaggregated) rabbit anti-human heavy- or light-chain sera, for 30 min at 4°C. Cells were washed twice in PBS and examined under UV, using a Zeiss Photomicroscope III equipped for epifluorescence microscopy. Positive scores were assessed as live cells showing bright surface-membrane staining as clear rings, caps or distinct speckling. To exclude background or non-specific fluorescence, cells were also incubated with the FITC-labelled second-layer antibody alone.

*E rosettes.*—These were performed using washed sheep erythrocytes according to methods described by Habeshaw *et al.* (1979). Positive E rosettes were scored as mononuclear leucocytes with 3 or more adherent sheep erythrocytes.

### RESULTS

*T lymphocytes*

The T-cell proportions found in blood and lymph nodes from patients and controls are detailed in Table III and IV. As defined by our results these tissues show a T-cell predominance in which E-rosetting ($Er^+$) and $OKT/Leu^+$ cells comprise 50–70% of all the mononuclear cells. The majority (70–85%) of T lymphocytes, thus defined, are comprised of cells expressing OKT4/Leu-3A (T "inducer") antigens. In the main, the proportions of cells expressing OKT1/Leu-1-reactive antigen are lower than either $OKT3^+$ cells or the sum of (OKT4/Leu-3A + OKT8/Leu-2A) but the differences are not significant. The percentages of $OKT3^+$ cells do, however, correlate well with the sum $(OKT4/Leu\text{-}3A+OKT8/Leu\text{-}2A)^+$ cells, *i.e.* T ("inducer" + "suppressor"). Differences in the relative proportions of $Er^+$ and pan-T antigen-expressing cells vary but are not significant. OKT6 antibody to thymic cortical cells was negative in all tissues.

The differences between the percentages of $Er^+$ cells in involved lymph nodes and "normal" controls are significant (Table III). There is also an apparently significant difference in the mean proportions of $OKT3^+$ cells between "normal" controls and diseased nodes but not in total T-cell proportions, defined by the sums of $(OKT4+8)^+$ and $(Leu\text{-}3A+2A)^+$ cells.

The surface-antigen profiles of mononuclear cells from "disease control" lymph nodes, do not differ significantly from those in involved nodes. However, there is a significant difference between mean OKT8/Leu-2A (T "suppressor") values in the "disease control" and "normal control" groups (18% and 11% respectively; $P=0{\cdot}01$).

No significant difference in results from patient and control blood has been demonstrated. The profiles are comparable to those from lymph nodes.

The data from spleen-cell suspensions however showed several differences (Table V): the mean totals of mononuclear cells expressing T-reactive antigens in spleens from patients with untreated HD, were lower than in either blood or lymph node ($P<0{\cdot}001$). They are accounted for by smaller relative proportions of OKT4/Leu-$3A^+$ cells.

The proportions of $Er^+$ cells in pre-treatment spleens equate with those in lymph node and blood, and it follows that the difference between the $Er^+$ and $OKT/Lev^+$ population, in spleen, is also significant ($P<0{\cdot}01$). In addition the

TABLE VI.—*Cell surface phenotypes in HD (% of total viable mononuclear-cell count)*

| Involved lymph nodes, by histological type | | E Rosettes | Pan T | OKT4/ Leu-3A | OKT8/ Leu-2A | T "helper"+ "suppressor" | Ia | SmIg |
|---|---|---|---|---|---|---|---|---|
| Lymphocyte predominance | No. obs. | 4 | 3 | 5 | 5 | 5 | 2 | 3 |
| | Mean | 59 | 67 | 55 | 10 | 65 | 15 | 21 |
| | s.e. | 4 | | 8 | 2 | 8 | — | — |
| Nodular sclerosis | No. obs. | 5 | 5 | 5 | 6 | 5 | 2 | 4 |
| | Mean | 61 | 69 | 55 | 21 | 76 | 16 | 23 |
| | s.e. | 4 | 2 | 8 | 3·5 | 7 | — | 5 |
| Mixed cellularity | No. obs. | 1 | 2 | 3 | 3 | 3 | 2 | 1 |
| | Mean | 62 | 78 | 59 | 8 | 67 | 20 | 12 |
| Lymphocyte depletion | 1 node | — | 29 | 15 | 7 | 22 | 44 | — |

T "inducer":"suppressor" ratio defined by OKT4/Leu-3A$^+$:OKT8/Leu-2A$^+$ cells approximates to 1:1, compared with 3–4:1, in blood and lymph node. Our data reveal no phenotypic differences between diseased and normal spleen. Phenotype profiles of 3 spleens obtained at laparotomy, after completion of chemotherapy, show decreased proportions of T cells, as defined by Er$^+$, OKT1, OKT3, Leu-1 and OKT4/Leu-3A$^+$ cells.

Data from lymph nodes were analysed according to the histological type of HD (Table VI). In lymph nodes involved with HD of lymphocyte-predominant type, there is an apparently greater predominance of T "inducer" (OKT4/Leu-3A) to T "suppressor" (OKT8/Leu-2A) lymphocytes (ratio 5:1). One node involved with HD of lymphocyte-depleted type shows an overall reduction in T lymphocytes, but with retention of T "helper" predominance. The numbers in each group are too small to allow statistical comparison.

*B lymphocytes*

The estimated proportions of cells defined either as surface-immunoglobulin-bearing cells (SIg$^+$) or HLA-DR (Ia$^+$) antigen-expressing cells, ranged from 12 to nearly 30%. Although there were some differences in the numbers counted by the two methods, these were not significant. When assessed by light-chain antisera, the B cells show a polyclonal profile.

*Controls*

All cells were strongly positive with W6/32 antibody. No positive reactions were seen with AN51, except with occasional platelet fragments which appeared as tiny, brilliantly fluorescing particles. Background fluorescence was almost always absent, apart from occasional faint, diffuse intracytoplasmic fluorescence.

### DISCUSSION

HD is characterized by the presence, in affected tissue, of high levels of "reactive" T lymphocytes. This could indicate either a marked antigenic stimulation by the neoplastic cell component of the lesion, or a persistent non-specific immune reaction. The frequently observed rise in serum immunoglobulins and circulating immune complexes (Madalinski *et al.*, 1970; Wagener *et al.*, 1976; Amlot *et al.*, 1978; Brown *et al.*, 1978) lends support to theories of general over-reactivity to chronic antigenic stimulation. On the other hand, a T inducer:suppressor imbalance in which the population of T cells consisted entirely or largely of inducer/helper cells, might lead to unbalanced and persistent inappropriate stimulation of B lymphocytes. In view of the abnormal cellular immunity so frequently observed, such lymphocytes might well be defective, either in stimulating or responding to antigenic challenge. Whilst our data demonstrate a T-cellpredominant ($Er^+$, $OKT1^+$, $OKT3^+$, $LEU\text{-}1^+$) cell population in diseased lymph nodes and peripheral blood from the patient population, the results are essentially identical to those obtained with the respective control tissues: uninvolved lymph nodes from the patients and normal donor blood.

Our results would appear to be at variance with an earlier study (Romagnani *et al.*, 1978) which showed an imbalance of subsets of peripheral-blood T-cells subsets in HD patients. Such cells identified as $Er^+$ cells expressing receptors for either IgM($T\mu$) or IgG ($T\gamma$), have been described by others (Moretta *et al.*, 1977) as subserving "helper"/"inducer" and "suppressor"/"cytotoxic" functions respectively. Romagnani was able to demonstrate a decrease in $T\mu$ and an increase in $T\gamma$ subsets though $T\mu$ lymphocytes remained overall predominant. Gupta & Tan (1980) demonstrated a small but non-significant increase in $T\gamma$ lymphocytes in the blood of children with untreated HD. These reports would thus suggest a relative increase in the "suppressor"/ "cytotoxic" T-cell population in the blood of HD patients. However, the methods for assaying these subpopulations involve reactions with quite different antigenic determinants from those recognized by the monoclonal antibodies, and it cannot be assumed that identical subsets are identified by the two methods.

Neither tonsils nor reactive lymph nodes from patients "cured" of HD might be regarded as appropriate control material for normal lymph nodes, and this reflects the difficulty in obtaining non-diseased lymph nodes, particularly from an age-matched group. Nonetheless, our data do show similar phenotype profiles, particularly in respect of the T "helper":"suppressor" ratios, though the overall degree of T-cell predominance is marginally less than that in lymph-node material from the patient population. The findings accord with previous reports of cell suspensions and frozen sections of normal tissues (Reinherz & Schlossman, 1980; Janossy *et al.*, 1980; Poppema *et al.*, 1981).

Because of the limited selection of histological types available for study, no conclusions can be drawn about possible T-cell differences in the 4 major histological types, though there is a suggestion of a higher proportion of T "inducer" lymphocytes in nodes involved with lymphocyte-predominant HD than in other subclasses. One lymph node from a patient aged 14, with mixed-cellularity HD, showed a T-helper population $>90\%$ of all cells, a marked predominance of this T subset. Since most of our patients presented with limited disease, it has not been possible to relate our findings to stage, or subsequent clinical progress.

Splenic T-cell populations may differ from those of lymph node or peripheral blood. A substantial proportion of $Er^+$ cells fails to express either pan-T antigens (OKT1, OKT3, Leu-1) or "helper"/ "inducer" antigens (OKT4, Leu-3A). This would imply the presence of an additional subset of T-cells, being either an immature population at an intermediate stage of differentiation, or a subset evolved in response to, or intrinsic to the disease process. Alternatively, it is conceivable that such a population could be resident in normal adult spleen. Gupta & Tan (1980) have demonstrated increased proportions of T-cells bearing IgM receptors in the spleens of their patients, who were, however, all children, mainly with advanced disease or splenic involvement.

The theory of "ecotaxopathy" proposed by De Sousa *et al.* (1976) implies a failure in HD of normal T-cell migration and recirculation (ecotaxis) with a preferential accumulation of these cells within the spleen. De Sousa's study of peripheral blood and spleen from 5 patients suggests that this abnormality is intrinsic to HD, and independent of stage or splenic involvement. Hunter *et al.* (1977) also demonstrated an overall increase of $Er^+$ cells in the spleens of patients, but most had advanced disease and heavy splenic infiltration, the highest

Er values being obtained from the involved spleens. In contrast, Payne *et al.* (1976) found the highest Er levels in uninvolved spleens, those in diseased spleens being within normal limits. Kaur *et al.* (1974) found normal levels of $Er^+$ cells in the spleens of HD patients, though responsiveness to PHA was increased.

We have not demonstrated an overall increase in $Er^+$ cells in the spleens of our patients although as discussed above these may well be distinct from those in peripheral nodes and blood. Only 1 patient, a 16-year-old female with nodular sclerosing HD, had heavy splenic involvement. Intriguingly, in this case the proportion of $Er^+$ cells was 78%, whilst 37% of the cells reacted with OKT1.

We conclude that our results, obtained from single-cell suspensions of lymph node, tonsil and peripheral blood, show phenotypic similarities in these tissues rather than any clear abnormalities. Compared with involved and uninvolved lymph nodes, there is a slightly lower T-cell proportion in the "normal" lymphoid control group. The proportional distribution of cells expressing "helper" and "suppressor" antigens is, however, very similar. The proportions of B cells defined by our methods do not vary significantly between tissues, and account for ~20% of leucocytes. A normal polyclonal profile is observed, findings similar to those of Bobrove *et al.* (1975).

The significance of the findings in the spleens of patients must remain speculative until appropriate numbers of normal controls for comparison are available.

Despite *in vivo* and *in vitro* demonstration of defective T-cell function in HD, this does not appear to result from deficiencies in the proportions of the T or B cells or T-subset ratios in diseased tissues. This suggests that some intrinsic functional immune deficiency is a significant feature in the pathogenesis of HD. Further work will attempt to clarify the micro-anatomical relationships of T and B subsets within the HD lesion, and to study some of the functional attributes of T cells from diseased tissues, in the hope of elucidating the nature of Hodgkin's disease.

We are grateful to Dr M. F. Greaves for his invaluable help, both in providing reagents and for advising on the design of this study. We also thank Professor J. S. Malpas for allowing us to study his patients and Dr A. G. Stansfeld of the Department of Histopathology who reviewed all the histological material. Our thanks also go to Jill Davey for typing the manuscript.

### REFERENCES

AISENBERG, A. C. (1966) Immunological status of Hodgkin's disease. *Cancer*, **19**, 385.

AMLOT, P. L., PRUSSELL, B., SLANEY, J. M. & WILLIAMS, B. D. (1978) Correlation between immune complexes and prognostic factors in Hodgkin's disease. *Clin. Exp. Immunol.*, **31**, 166.

BECTON DICKINSON (1981) FACS systems monoclonal antibody centre, *Source Book Sections* 4.1, 2, 3.

BOBROVE, A. M., FUCKS, Z., STROBER, S. & KAPLAN, H. S. (1975) Quantitation of T and B lymphocytes and cell mediated immunity in Hodgkin's disease. *Cancer*, **36**, 169.

BRODSKY, F. M., PARHAM, P., BARNSTABLE, C. J., CRUMPTON, M. J. & BODMER, W. F. (1979) Hybrid myeloma monoclonal antibodies against MHC products. *Immunol. Rev.*, **47**, 3.

BROWN, C. A., HALL, C. L., LONG, J. C., CAREY, K., WEITZMAN, S. A. & AISENBERG, A. C. (1978) Circulating immune complexes in Hodgkin's disease. *Am. J. Med.*, **64**, 289.

CARBONE, P. P., KAPLAN, H. S., MUSSHOFF, K., SMITHERS, D. W. & TUBIANA, M. (1971) Report of the Committee on Hodgkin's Disease Staging Classification. *Cancer Res.*, **31**, 1860.

DE SOUSA, M., YANG, M., LOPES-CORRALES, E. & 4 others (1977) Ecotaxis: The principle and its application to the study of Hodgkin's disease. *Clin. Exp. Immunol.* **27**, 143.

DUTTON, R. W. (1975) Suppressor T cells. *Transplant Rev*, **26**, 39.

ELTRINGHAM, J. R. & KAPLAN, H. S. (1973) Impaired delayed hypersensitivity responses in 154 patients with untreated Hodgkin's disease. *Nat Cancer Inst. Monog*, **36**, 107.

EVANS, R. L., LAZARUS, H., PENTA, A. C. & SCHLOSSMAN, S. E. (1978) Two functionally distinct subpopulations of human T cells that collaborate in the generation of cytotoxic cells responsible for cell mediated lympholysis. *J. Immunol.*, **129**, 1423.

GUPTA, S. & TAN, C. (1980) Subpopulations of human T lymphocytes. XIV. Abnormality of T cell locomotion and of distribution of subpopulations of T and B lymphocytes in peripheral blood and spleen from children with untreated Hodgkin's disease. *Clin. Immunol. Immunopathol.*, **15**, 133.

HABESHAW, J. A., CATLEY, P. F., STANSFELD, A. G. & BREARLEY, R. L. (1979) Surface phenotyping, histology and the nature of non-Hodgkin's lymphoma in 157 patients. *Br. J. Cancer*, **40**, 11.

HOFFMAN, R. A., KUNG, P. C., HANSEN, W. P. & GOLDSTEIN, G. (1980) Simple and rapid measurement of human lymphocytes T and their subclasses in peripheral blood. *Proc. Natl Acad. Sci.*, **77**, 4914.

HUNTER, C. P., PINKUS, G., WOODWARD, L., MOLONEY, W. C. & CHUVCHILL, W. H. (1977) Increased T lymphocytes and Ig MEA-receptor lymphocytes in Hodgkin's disease spleens. *Cell Immunol.*, **31**, 193.

JACKSON, H. JR. & PARKER, F. JR. (1947) *Hodgkin's Disease and Allied Disorders*. New York: Oxford University Press.

JANOSSY, G., TIDMAN, N., SELBY, W. S. & 4 others (1980) Human T lymphocytes of inducer and suppressor type occupy different micro-environments. *Nature*, **288**, 81.

KAUR, J., SPIERS, A. S. D., CATOVSKY, D. & GALTON, D. A. G. (1974) Increase of T lymphocytes in the spleen in Hodgkin's disease. *Lancet*, ii, 800.

KUNG, P., GOLDSTEIN, G., REINHERZ, E. L. & SCHLOSSMAN, S. F. (1979) Monoclonal antibodies defining distinctive human T cell surface antigens. *Science*, **206**, 347.

LEVY, R. A. & KAPLAN, H. S. (1974) Impaired lymphocyte function in untreated Hodgkin's disease. *N. Engl. J. Med.* **290**, 181.

LUKES, R. B. & BUTLER, J. J. (1966) The pathology and nomenclature of Hodgkin's disease. *Cancer Res.*, **26**, 1063.

MCMICHAEL, A. J., RUST, N. A., PLICH, J. R. *et al.* (1982) Monoclonal antibody to human platelet glycoprotein II: Immunological studies. *Br. J. Haematol.* (in press).

MADALINSKI, K., BROZOSKO, W. S. & SULAWSKI, M. (1970) Immunoglobulin levels in Hodgkin's disease. *Haematologia, (Budap)*, **4**, 333.

MORETTA, L., WEBB, S., GROSSI, C. E., LYDYARD, P. M. & COOPER, M. D. (1977) Functional analysis of two human lymphocyte subpopulations: Help and suppression of B cell responses by T cells bearing receptors for IgM or IgG. *J. Exp. Med.*, **146**, 184.

PARKER, F. JR., JACKSON, H. JR., FITZHUGH, G. & SPIES, T. D. (1932) Studies of disease of the lymphoid and myeloid tissues. IV. Skin reactions to human and avian tuberculin. *J. Immunol.*, **22**, 277.

PAYNE, S. V., JONES, D. B., HAGGERT, D. G., SMITH, J. L. & WRIGHT, D. H. (1976) T and B lymphocytes and Reed-Steinberg Cells in Hodgkin's disease lymph nodes and spleens. *Clin. Exp. Immunol.*, 24, 280.

POPPEMA, S., NHAN, A. K., REINHERZ, E. L., MCCLUSKY, R. T. & SCHLOSSMAN, S. F. (1981) Distribution of T cell subsets in human lymph nodes. *J. Exp. Med.*, **153**, 30.

REED, D. M. (1902) On the pathological changes in Hodgkin's disease, with especial reference to tuberculosis. *Johns Hopkins Hosp. Rep.*, **10**, 133.

REINHERZ, E. L., KUNG, P. C., GOLDSTEIN, G. & SCHLOSSMAN, S. F. (1979*a*) Further characterisation in the human inducer T cell subset, defined by *J. Immunol.*, **123**, 2894.

REINHERZ, E. L., KUNG, P. C., GOLDSTEIN, G. & SCHLOSSMAN, S. E. (1979*b*) A monoclonal antibody with selective reactivity with functionally mature human thymocytes and all peripheral human T cells. *J. Immunol.*, **123**, 1312.

REINHERZ, E. L., KUNG, P. C., GOLDSTEIN, G. & SCHLOSSMAN, S. F. (1980) A monoclonal antibody reactive with human cytotoxic/suppressor subset previously defined by a heteroantiserum termed $TH_2$. *J. Immunol.*, **124**, 1301.

REINHERZ, E. L. & SCHLOSSMAN, S. F. (1980) The differentiation and function of human T lymphocytes. *Cell*, **19**, 821.

ROMAGNANI, S., MAGGI, E., BIAGIOTTI, R., GUIDIZI, M. G., AMADORI, A. & RICCI, M. (1978) Altered proportion of the $T\mu$ and $T\gamma$ cell subpopulations in patients with Hodgkin's disease. *Scand. J. Immunol.*, **7**, 511.

ROWLEY, D. D., FILETH, F. W., STUART, F. P., KOHLER, H. & COSENZA, H. (1973) Specific suppression of immune responses. *Science*, **181**, 1133.

SCHIER, W., ROTH, A., OSTROFF, G. & SCHRIFT, M. H. (1956) Hodgkin's disease and immunity. *Am. J. Med.*, **20**, 94.

SOKAL, J. E. & PRIMIKIRIOS, M. (1961) The delayed skin test response in Hodgkin's disease and lymphosarcoma. *Cancer*, **14**, 597.

SOKAL, J. E. (1973) Immunologic deficiency in Hodgkin's disease. *Tumori.*, **59**, 343.

STEINER, P. E. (1934) Aetiology of Hodgkin's disease. Skin reactions to avian and human tuberculin proteins in Hodgkin's disease. *Arch. Int. Med.*, **54**, 11.

WAGENER, D. J. T., VAN MEINSTER, P. J. J. & HAANEN, C. (1976) The immunoglobulins in Hodgkin's disease. *Eur. J. Cancer*, **12**, 683.

WEBB, D. R. JR. & JAMIESON, A. T. (1976) Control of mitogen-induced transformation: Characteristics of a splenic suppressor cell and its mode of action. *Cell. Immunol.*, **24**, 45.

YOUNG, R. C., CORDER, M. P., BERARD, C. W. & DE VITA, V. T. (1973) Immune alterations in Hodgkin's disease. Effect of delayed hypersensitivity and lymphocyte transformation on course and survival. *Arch. Int. Med.*, **131**, 446.